# Massive non-functioning Adrenocortical carcinoma presenting as acute abdomen without rupture: A case report

**DOI:** 10.1016/j.eucr.2026.103350

**Published:** 2026-01-19

**Authors:** Shady H.Y.B. Girgis, Ihab H.Y. Barsoum, Shady Elia Anis, Mostafa Elgamal, Hany Yassa

**Affiliations:** a Urology Department, Air Force Specialized Hospital, Cairo, Egypt; b Pathology Department, Kasr Alainy School of Medicine, Cairo University, Cairo, Egypt; c Urology Department, Forth Valley Royal Hospital NHS Trust, Scotland, UK

**Keywords:** Adrenocortical carcinoma, Case report, Acute abdomen, Non-functioning tumor

## Abstract

Adrenocortical carcinoma (ACC) is a rare, aggressive malignancy with diverse clinical presentations. Acute abdomen is an exceptionally uncommon manifestation, typically reflecting tumor rupture. We report a 39-year-old male presenting with acute abdominal pain and a palpable left upper-quadrant mass. Imaging demonstrated a massive adrenal tumor (17.8 × 15.1 × 20.2cm). En-bloc resection with negative margins was achieved without rupture while preserving adjacent organs. Histopathology confirmed ACC. The patient remains disease-free 36 months postoperatively without adjuvant therapy. Acute abdomen can be the presenting symptom of ACC even without tumor rupture. Timely intervention can prevent possible impending rupture, enabling complete resection and favorable oncological outcomes.

## 1 Introduction

Adrenocortical carcinoma (ACC) is an uncommon malignancy of the adrenal cortex, with an estimated incidence and prevalence of 0.7–2 and 4–12 cases per million/year worldwide, respectively.^1^^,^^2^ Coupled with its rarity, ACC carries a poor prognosis and is one of the most aggressive endocrine tumors, second only to anaplastic thyroid carcinoma.^3^^,^^4^ ACC accounts for approximately 0.2 % of all cancer-related deaths in the United States. The disease exhibits a bimodal age distribution, with incidence peaks in the first and then fourth to fifth decades of life.^5^

The clinical manifestations of ACC vary depending on the hormonal functionality of the tumor as well as its size. Approximately 60 % of ACCs are hormone-secreting, most often associated with hypercortisolism (Cushing syndrome), virilization, or a mixed presentation. In contrast, non-functioning tumors are typically detected later, either incidentally during imaging for other indications or due to abdominal mass effects.^6^ This often leads to delayed diagnosis and potentially poorer long-term outcomes.^7^ Radiologically, ACCs appear as large, heterogeneous adrenal masses with features such as necrosis, hemorrhage, and occasional calcifications.^8^

Acute abdomen is a rare initial presentation of ACC and is usually attributed to tumor rupture and intra-abdominal hemorrhage.^9^^,^^10^ Reports of acute abdomen caused solely by the mass effect of ACC without rupture are extremely scarce. In the following case report, we present a rare case of a massive, non-functioning ACC presenting with acute abdomen without rupture, successfully managed with margin-negative resection and a tumor-free long-term surveillance.

## 2 Case presentation

A 39-year-old male patient presented to the emergency department with an acute exacerbation of abdominal pain on top of a 2-month history of progressive left loin pain and discomfort. The pain was described as sharp, exacerbated with movement, and rated 9/10 on the visual analog scale. The patient denied any nausea, vomiting, bowel habit changes, or urinary symptoms. His past medical history was unremarkable, apart from smoking. There was no alcohol consumption, unconventional environmental exposures, or relevant family history of endocrine or genetic syndromes.

On initial presentation, the patient's temperature was 37.6 °C, with mild tachypnea and tachycardia of 21 breaths/min and 108 beats/min, respectively. His blood pressure was 100/70 mmHg. Clinically, the patient exhibited mildly noticed general manifestations; his skin was cold, clammy, and sweaty, with generalized weakness and lethargy. On physical examination, his abdomen was slightly rigid with moderate tenderness and rebound tenderness. It also revealed a huge, palpable mass occupying the whole left upper quadrant and extending into the lower left quadrant of the abdomen. Other systemic examinations were unremarkable.

Initial laboratory investigations revealed mild anemia with a hemoglobin (Hb) level of 11.8 g/dL (Reference: 13.0–18.0 g/dL), leukocytosis with a total leukocytic count (TLC) of 13.4 × 10^9^/L (Reference: 4.0–11.0 × 10^9^/L), and metabolic acidosis on arterial blood gas (ABG) analysis. His liver and renal function tests, coagulation profile, cardiac enzymes, and electrolytes were all within normal limits. Initial endocrine evaluation, including serum cortisol, aldosterone, renin, dehydroepiandrosterone sulfate (DHEA-S), adrenocorticotropic hormone (ACTH), free testosterone, and urine metanephrines were all within normal ranges. Also, an electrocardiogram (ECG) showed a normal sinus rhythm.

**Fig. 1:**
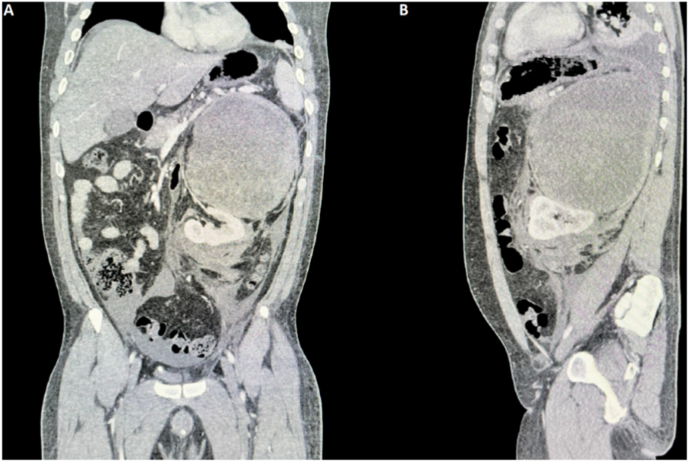
Abdominopelvic triphasic CT scan. A: Coronal view demonstrating the inferomedial displacement of the left kidney and its pedicle. B: Sagittal view.

**Fig. 2:**
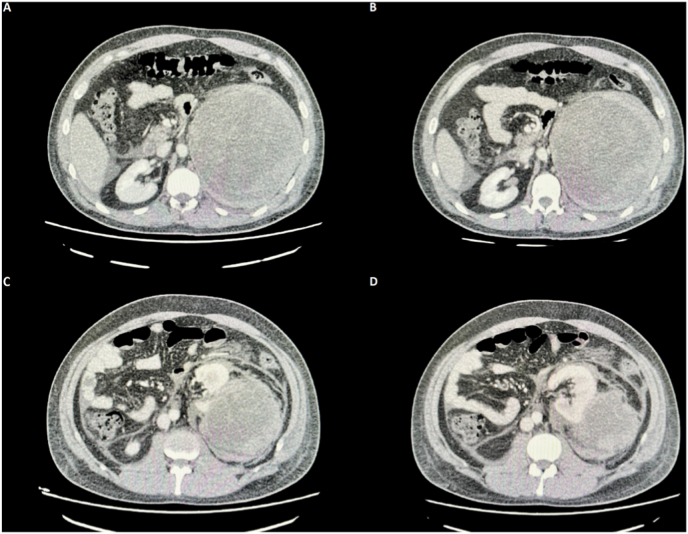
Abdominopelvic triphasic CT scan at different axial sections. Showing the tumor at multiple levels, denoting its maximum diameter, the edging towards the midline, and its proximity to the left kidney.

Radiologically, a pelviabdominal ultrasound revealed a sizeable, 15 cm × 13 cm hyperechoic lesion occupying the left lienorenal space with displacement of the left kidney. A subsequent triphasic computerized tomography (CT scan) revealed a massive, heterogeneous, thick-walled left adrenal mass measuring 17.8×15.1×20.2cm. The lesion was close to crossing the midline, displacing the left kidney with its pedicle inferomedially towards the midline, without any visible evidence of invasion to surrounding organs or vascular structures. There were no associated visible lymphadenopathies or distant metastases (Fig. 1, Fig. 2). MRI also confirmed similar findings.

Consequently, the likely provisional diagnosis was a huge suprarenal tumor, most likely a non-functioning ACC.

The patient was then admitted, stabilized, and his pain was managed adequately. He was closely monitored and prepared with the cooperation of a multidisciplinary team for an open resection and excision of the lesion, with the possibility of undergoing a left radical or partial nephrectomy depending on the intraoperative assessment of the left kidney.

**Fig. 3:**
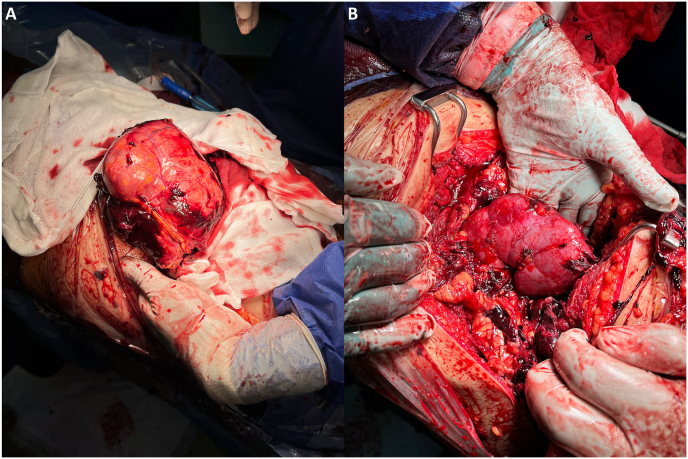
Intraoperative views. A: Showing the tumor during its deliverance. B: The spared left kidney after successful complete tumor excision.

The surgical procedure started with an extended left lumbar incision exposing the mass. After its careful dissection from the surrounding structures, an en-bloc excision and delivery of the mass was successful (Fig. 3-A). The spleen, pancreas, and left kidney were obviously displaced from their conventional anatomical positions. Fortunately, surgical margins were grossly negative intraoperatively, and the surrounding tissues were not invaded. Henceforth, there was no need to perform the nephrectomy, and the left kidney was preserved (Fig. 3-B). Other surrounding organs were spared as well, and no visibly enlarged lymph nodes were detected intraoperatively either. However, hilar lymph nodes were excised for pathological evaluation.

The patient was then transferred back to the ICU for postoperative care and managed there for the next two days. On the first postoperative day, the patient started complaining of weakness and numbness affecting the right upper limb. Investigations requested from the consulted neurology, neurosurgery, and vascular surgery departments all opted to confirm that it was due to a neuropraxia affecting his right proximal brachial plexus, which was managed conservatively and fully resolved three weeks after hospital discharge. Otherwise, the postoperative period passed uneventfully, the surgical drain was removed, and the patient was discharged from the hospital on the 4th postoperative day.

**Fig. 4:**
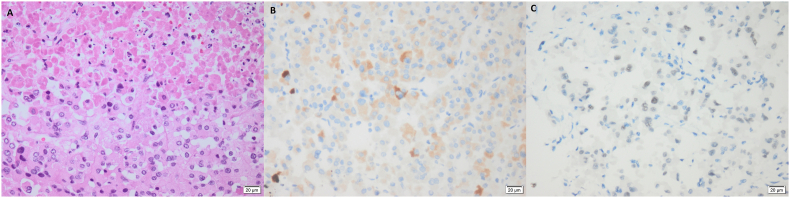
**Histopathological and Immunohistochemical assessments (x400).** A: tumor section showed large polyhedral cells having ill-defined cell borders with abundant eosinophilic granular cytoplasm, pleomorphic hyperchromatic nuclei, and confluent necrosis. B: Tumor cells with focal cytoplasmic expression for Melan A. C: Tumor cells showing focal nuclear expression for Steroidogenic factor 1 (SF-1).

Originating from the left adrenal gland, the histopathological examination of the excised mass confirmed the diagnosis of ACC. Microscopically, the tumor showed polyhedral epithelial-type cells with abundant granular eosinophilic cytoplasm with ill-defined cell borders - with no clear cell foci - arranged in trabeculae, nests, and solid sheets (50 % diffuse architecture) with variable degrees of nuclear atypia (ranging from mild to marked) and 70 % confluent tumoral necrosis. Mitotic index 2/50HPF was also noted with no detectable abnormal mitotic forms. No capsular, vascular, or sinusoidal invasions were seen (Fig. 4-A). Additionally, the excised lymph nodes did not show any malignant cell infiltration.

Immunohistochemical results revealed tumor cells’ cytoplasmic expression for Pan-CK, Synaptophysin, Calretinin, Melan A, and nuclear expression for Steroidogenic Factor-1 (SF-1) (Fig. 4-B and C).

Collectively, these findings denoted a Weiss score of 4 and a modified Weiss score of 3, positively confirming the malignancy.

**Fig. 5:**
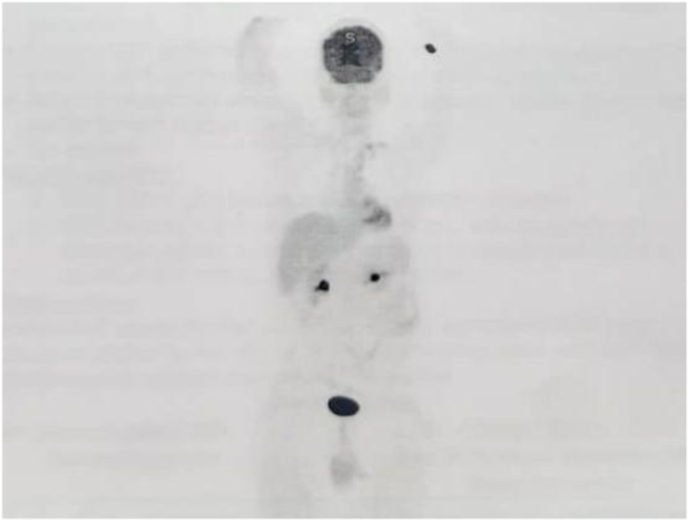
18F-fluorodeoxyglucose positron emission tomography (FDG–PET). Done on the latest follow-up appointment denoting a clear study, with the absence of any local recurrence or distant metastases.

At 36 months postoperatively, the patient is doing well, both physically and psychologically, and is regularly attending follow-up appointments with the oncology and urology outpatient clinics. The team did not initiate any adjuvant therapy postoperatively. The most recently performed 18 F-fluorodeoxyglucose positron emission tomography (FDG–PET) revealed a completely normal study, with no evidence of distant metastases nor locoregional recurrences (Fig. 5).

## 3 Discussion

ACC is a rare and highly aggressive malignancy. Its presentation as an acute abdomen is particularly uncommon, usually occurring after tumor rupture.^9^^,^^10^ Our case is unique in that the acute abdomen resulted exclusively from mass effect, without any radiologic or operative evidence of tumor rupture. Additionally, the initial clinical picture included initial, subtle manifestations of hemodynamic instability that were accompanied by a normal hormonal profile, raising initial concerns that an impending rupture of the non-functioning adrenal mass might have been imminent. Fortunately, this was not the case. The most probable cause of the acute abdomen, in this case, was probably due to the abdominal mass effect exerted by the aggressive growth pattern that the mass exhibited, considerably compressing the surrounding neighboring structures.

A limited number of publications depicted cases with ACC that presented with an acute abdomen. In total, 19 similar cases have been documented in the literature; 16 of these were reviewed and summarized by Polistina et al.^11^ with three additional cases recently reported by Kashiwagi et al.,^7^ Khan et al.,^12^ and Yoshida et al.^13^ Across the 19 ACC cases that presented with acute abdominal pain, all showed radiological and/or intraoperative evidence of tumor rupture, and - except a single case - all required immediate surgical intervention ^7^^,^^11^, ^12^, ^13^. Our case adds to this limited literature by demonstrating that an acute abdomen may occur in massive ACC without rupture.

The European Network for the Study of Adrenal Tumors (ENSAT) classification for ACC staging is commonly used. It stratifies ACCs into four stages. Stage I and II entail tumor sizes of ≤5cm and >5cm, respectively, and neither of these two stages has any positive lymph nodes or distant metastases (Stage I = TNM: T1 N0 M0, Stage II = TNM: T2 N0 M0). Stage III describes a locally invasive ACC regardless of its size. Such an infiltration could be in the form of a local spread to the surrounding tissues, a tumor thrombus/invasion of the renal vein or the IVC, or positive lymph nodes. This stage is equivalent to a TNM staging of T1–2 N1 M0 or T3–4 N0–1 M0. Finally, stage IV denotes the presence of distant metastases (TNM: T1-4 N0-1 M1).^1^ Given the huge size of the tumor (>5cm), negative lymph nodes, and the absence of any distant metastases, a stage II ACC was assigned to the presented case's diagnosis. Regarding Prognosis, stage II tumors' 5-year survival rate is 63 %.^14^ With margin-free complete resection (R0 resection), the median survival duration is 74 months, while dropping to a median of 6–27 months of survival with incomplete resection.^15^ This underscores the importance of a rapid diagnosis and management in such cases to avoid the adverse consequences of tumor rupture and the potential for poorer oncological outcomes, particularly with the aggressive growth patterns of ACC.^1^

To confirm the diagnosis of ACC, the retrieved mass should undergo a histopathological analysis. Entailing nine different criteria, the Weiss scoring system is a well-established method for pathological diagnosis. A Weiss score of three or more denotes a malignant tumor.^16^ Upon assessment, the excised tumor fulfilled four of them, which are: Confluent necrosis, marked nuclear atypia, 50 % diffuse architecture, and almost total absence of clear cells. On the other hand, the modified Weiss scoring system closely resembles the original Weiss system, yet the former consists of only five criteria to be assessed, with a slightly different scoring layout. However similarly, a score of three or more is also needed to confirm malignancy.^16^ Given the previously mentioned histopathological features of the excised tumor, a modified Weiss score of three has been given.

Postoperative surveillance should be maintained every three months for the first two years since surgery, including both a radiological investigation and hormonal profiling, as the latter serves as a biomarker for possible recurrence.^1^ Thereafter, follow-up should continue at a decreasing frequency for a minimum of ten years.^1^^,^^5^ Regarding our patient, he has been regularly attending the follow-up appointments and has remained tumor-free for 36 months postoperatively. This was confirmed by the most recent FDG–PET, which showed no evidence of any recurrence, in addition to the normal hormonal profiles, all while the oncology team not offering him any form of adjuvant chemotherapy or radiotherapy after surgery.

## 4 Conclusion

We report an uncommon case of a massive, non-functioning ACC with the rare presentation of acute abdomen in the absence of tumor rupture. The case emphasizes the importance of maintaining a high index of suspicion to enable timely diagnosis and management of these cases, mitigating the risk of a possible impending rupture and allowing a better, margin-negative surgical excision that remains a cornerstone for better oncological outcomes.

## CRediT authorship contribution statement

**Shady H.Y.B. Girgis:** Conceptualization, Data curation, Formal analysis, Investigation, Methodology, Project administration, Writing – original draft, Writing – review & editing. **Ihab H.Y. Barsoum:** Conceptualization, Data curation, Formal analysis, Writing – original draft, Writing – review & editing. **Shady Elia Anis:** Conceptualization, Data curation, Formal analysis, Investigation, Methodology, Writing – original draft, Writing – review & editing. **Mostafa Elgamal:** Conceptualization, Data curation, Formal analysis, Investigation, Methodology, Writing – original draft, Writing – review & editing. **Hany Yassa:** Conceptualization, Data curation, Formal analysis, Investigation, Methodology, Project administration, Supervision, Validation, Writing – review & editing.

## Ethical approval and consent to participate

Informed written consent was provided by the patient to participate in this study and for its publication. Also, the approval of the research ethical committee of the Air Force Specialized Hospital has been obtained as well. Both documents are available upon request.

## Consent for publication

Consent for publication has been obtained from the patient regarding the data included in the case report and associated images. In case needed, a copy of the consent form is available to be reviewed by the Editor-in-Chief of this journal.

## Availability of data and materials

Not applicable to this study since no datasets have been generated or analyzed throughout its process.

## Clinical trial number

Not Applicable.

## Funding

This research did not receive any specific grant from funding agencies in the public, commercial, or not-for-profit sectors.

## Declaration of competing interest

The authors declare that they have no known competing financial interests or personal relationships that could have appeared to influence the work reported in this paper.

## List of Abbreviations:

ACC: Adrenocortical carcinoma
ACTH: Adrenocorticotropic hormone
ABG: Arterial blood gases
CT: Computerized tomography
DHEA-S: Dehydroepiandrosterone sulfate
ECG: Electrocardiogram
ENSAT: European Network for the Study of Adrenal Tumors
FDG–PET: Fluorodeoxyglucose positron emission tomography
Hb: Hemoglobin
ICU: Intensive care unit
MRI: Magnetic resonance imaging
SF-1: Steroidogenic factor-1
TLC: Total leucocytic count
